# Regional Variation in Antioxidant and Anti-Inflammatory Activities of the Brown Alga *Sargassum thunbergii* and Mechanistic Role of Fucosterol in Inflammation Modulation

**DOI:** 10.3390/biomedicines13112808

**Published:** 2025-11-18

**Authors:** Sung-Gyu Lee, Hyun Kang

**Affiliations:** 1 Department of Medical Laboratory Science, College of Health Science, Dankook University, Cheonan-si 31116, Chungcheongnam-do, Republic of Korea; 2 Department of Medical Laboratory Science, Marine Bio-Food and Drug Convergence Technology Center, Dankook University, Cheonan-si 31116, Chungcheongnam-do, Republic of Korea

**Keywords:** *Sargassum thunbergii*, fucosterol, antioxidant activity, iNOS inhibition, molecular docking, functional food ingredients, natural drug discovery

## Abstract

**Background/Objectives:** Natural products derived from marine algae serve as promising reservoirs of bioactive compounds for preventing and managing inflammation-associated diseases. This study systematically investigated the regional variations in antioxidant and anti-inflammatory activities of the brown alga *Sargassum thunbergii* collected from seven coastal regions of Korea and elucidated the underlying mechanisms of action. **Methods:** Among all samples, the extract from Haeundae-gu exhibited the highest total polyphenol (47.3 ± 2.1 mg GAE/g) and flavonoid (19.8 ± 1.7 mg QE/g) contents, showing superior antioxidant activity in 2,2-diphenyl-1-picrylhydrazyl (DPPH) (RC_50_ = 44.54 µg/mL), 2,2′-Azino-bis(3-ethylbenzothiazoline-6-sulfonic acid) (ABTS) (RC_50_ = 36.42 µg/mL), and ferric reducing antioxidant power (FRAP) (0.56 mM FeSO_4_ eq/mg) assays. In lipopolysaccharide (LPS)-stimulated RAW 264.7 macrophages, the Haeundae-gu extract markedly suppressed nitric oxide (NO) production and downregulated inducible nitric oxide synthase (iNOS) expression in a dose-dependent manner without cytotoxic effects. High-performance liquid chromatography (HPLC) identified fucosterol (10.23 ± 0.17 mg/g extract) as a predominant sterol component, and molecular docking analysis revealed specific hydrogen bonding of fucosterol with TYR489 in the active site of iNOS (GlideScore = −4.774 kcal/mol). **Results:** These findings indicate that both phenolic compounds and sterols such as fucosterol act synergistically to enhance the antioxidant and anti-inflammatory effects of *S. thunbergii*. **Conclusions:** In summary, the mechanistic and functional insights obtained in this study highlight *S. thunbergii*, particularly from Haeundae-gu, as a promising marine-derived bioresource for developing nutraceuticals and therapeutic interventions against oxidative stress and inflammation-related disorders.

## 1. Introduction

Natural products from marine organisms represent an invaluable reservoir of structurally diverse and pharmacologically active molecules with significant therapeutic potential. Among marine resources, brown macroalgae (*Phaeophyceae*) have attracted particular attention as rich sources of secondary metabolites such as phlorotannins, fucoidans, and meroterpenoids, which exhibit potent antioxidant, anti-inflammatory, and metabolic regulatory activities [1,2,3].

*Sargassum thunbergii* (*S. thunbergii*), a brown alga widely distributed along the Korean coastline, has been extensively investigated for its pharmacological potential, particularly its ability to modulate oxidative stress and inflammation [4,5]. Phytochemical and metabolomic investigations have revealed various bioactive constituents, including fucoidan-type polysaccharides, phytosterols (notably fucosterol, isofucosterol, and saringosterol), carotenoids such as fucoxanthin, and meroterpenoids known as thunbergols [6,7,8]. Among these, fucosterol has emerged as a predominant sterol with antioxidant, anti-inflammatory, and lipid-lowering properties, suggesting its potential utility as a pharmacologically relevant scaffold for anti-inflammatory drug discovery [6,8].

However, the biochemical composition and biological efficacy of *S. thunbergii* are strongly influenced by environmental parameters such as water temperature, salinity, light intensity, and anthropogenic stress, all of which can modulate secondary metabolite biosynthesis and bioactivity [9]. Therefore, understanding and quantifying this biochemical variation is essential for establishing standardized marine bioresources suitable for reliable therapeutic and functional applications. Chronic diseases, including cardiovascular, neurodegenerative, and metabolic disorders, are closely associated with oxidative stress–induced cellular damage [10,11]. Natural antioxidants from marine algae are increasingly recognized as key modulators of reactive oxygen species (ROS), helping to maintain cellular redox balance and protect against inflammation-mediated tissue injury [12].

Inflammation, while vital for host defense, can become detrimental when persistently activated, contributing to the pathogenesis of chronic diseases such as arthritis, inflammatory bowel disease, and metabolic syndromes [13]. Marine-derived polyphenols and polysaccharides have been shown to suppress inflammatory cascades by downregulating pivotal mediators, including nuclear factor-kappa B (NF-κB), tumor necrosis factor-alpha (TNF-α), and inducible nitric oxide synthase (iNOS) [14,15]. Consistent with these findings, *S. thunbergii* exhibits notable antioxidant and anti-inflammatory properties, highlighting its potential as a marine-derived bioresource for the development of nutraceuticals and therapeutics targeting inflammation-associated disorders [16].

Despite increasing evidence, integrated investigations connecting environmental variation with biochemical composition and functional bioactivity in brown algae remain limited. Environmental factors such as temperature, salinity, and light availability profoundly affect the biosynthesis of phlorotannins and other phenolic metabolites, which ultimately determine antioxidant and anti-inflammatory efficacy. Kamiya et al. [17] demonstrated that *Sargassacean* species from the coast of the Sea of Japan exhibit pronounced seasonal variation in phlorotannin content, revealing how habitat conditions shape metabolic composition and biological efficacy. On a broader scale, Kumar et al. [18] emphasized that environmental changes—from local to global levels—modulate algal metabolism both directly and through ecological interactions, highlighting the complex interplay between environmental stressors and functional traits in brown macroalgae.

Moreover, recent advances in molecular docking and in silico modeling have provided powerful tools to elucidate the molecular mechanisms of marine natural products, offering deeper insight into their potential as anti-inflammatory drug leads. Structure-based computational approaches have been applied to marine-derived phenolic compounds such as phlorotannins to predict their binding affinity toward viral and inflammatory targets. For example, Kang et al. [19] performed in silico screening of marine phlorotannins and identified strong-binding candidates with pharmacological versatility beyond antioxidant activity. Likewise, Kumari et al. [20] employed in silico strategies to evaluate pharmacological constraints of natural compounds against key inflammatory enzymes such as cyclooxygenase-2 (COX-2) and 5-lipoxygenase (5-LOX), reinforcing the utility of computational modeling in anti-inflammatory drug discovery. Building upon these precedents, this study specifically applies molecular docking to reveal the precise inhibitory mechanism of *S. thunbergii*-derived fucosterol. By quantifying its binding affinity and detailing the structural interactions with key inflammatory effectors, iNOS, we provide the first direct mechanistic evidence that complements existing cellular data and validates fucosterol’s potential as a targeted anti-inflammatory lead.

To the best of our knowledge, this study is the first to systematically compare *S. thunbergii* collected from seven distinct Korean coastal regions, integrating biochemical, biological, and computational analyses. By linking environmental variability to biochemical composition and functional bioactivity, and by identifying fucosterol–target interactions through molecular docking, this work provides both empirical and mechanistic insights into the therapeutic potential of *S. thunbergii*. The outcomes of this research are expected to advance the understanding of marine algal bioresources as sustainable platforms for developing functional ingredients and natural anti-inflammatory agents applicable to both nutraceutical and pharmaceutical industries.

## 2. Materials and Methods

### 2.1. Sample Acquisition and Extraction

**Figure 1:**
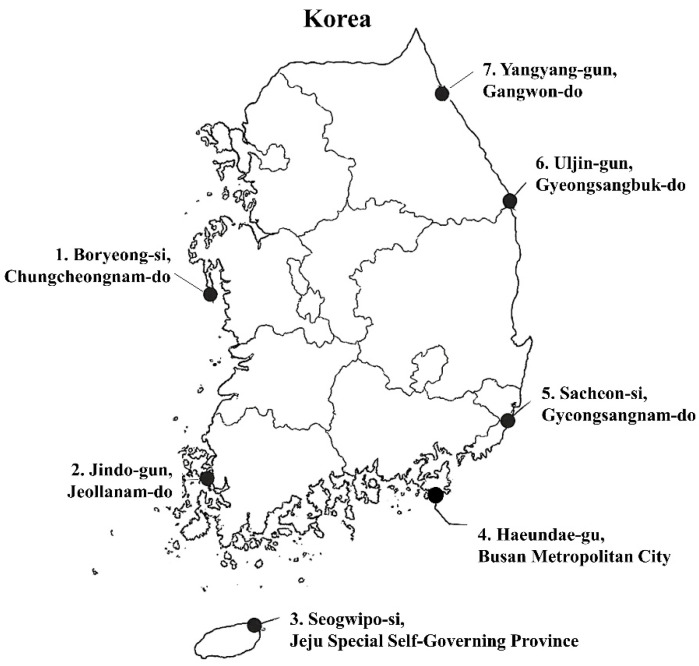
Geographical distribution of *S. thunbergii* collection sites along the coastal regions of Korea. The map highlights the seven sampling locations: Boryeong-si (Chungcheongnam-do), Jindo-gun (Jeollanam-do), Seogwipo-si (Jeju Special Self-Governing Province), Haeundae-gu (Busan Metropolitan City), Sacheon-si (Gyeongsangnam-do), Uljin-gun (Gyeongsangbuk-do), and Yangyang-gun (Gangwon-do). Samples were provided by the National Marine Biodiversity Institute of Korea (MABIK) under its biological resource distribution program. Created with ChatGPT (GPT-5) image generation tool, OpenAI ChatGPT-5.1 (San Francisco, CA, USA; accessed on 8 Setember 2025).

*S. thunbergii* extracts used in this study were provided by the National Marine Biodiversity Institute of Korea (MABIK, Seocheon, Republic of Korea) under its biological resource distribution program. The samples were collected during the summer vegetative growth phase (June–July) from seven different coastal regions in Korea (Table 1, Figure 1) to represent diverse ecological environments across the east, west, and south coasts, Jeju Island, and an urbanized coastal site. Upon collection, the samples were rinsed with local seawater, washed thoroughly with distilled water, air-dried under shade for 48 h, lyophilized, and then pulverized into fine powder (40-mesh). For extraction, 10 g of powdered sample was mixed with 200 mL of 70% ethanol (1:20, *w*/*v*). Ultrasonic-assisted extraction was performed at 40 kHz and 250 W for 60 min at 40 °C. The extracts were filtered, re-extracted twice under identical conditions, pooled, and concentrated under reduced pressure at 40 °C using a rotary evaporator. The concentrated extracts were lyophilized, stored at −20 °C until further use, and analyzed directly in the experiments [21].

### 2.2. Total Polyphenol and Flavonoid Contents

#### 2.2.1. Total Polyphenol Content (TPC) Assay

The total polyphenol content (TPC) of each extract was determined using a modified Folin–Denis method [22]. Briefly, 1 mL of each sample solution (1 mg/mL) was mixed with 1 mL of a twofold-diluted Folin–Ciocalteu’s phenol reagent (Sigma-Aldrich, St. Louis, MO, USA) and incubated at room temperature (RT) for 3 min. Subsequently, 1 mL of 10% sodium carbonate solution (Na_2_CO_3_, Sigma-Aldrich) was added, and the mixture was left at RT for 1 h. The absorbance was read at 700 nm using a microplate spectrophotometer (xMark™, Bio-Rad, Hercules, CA, USA). Gallic acid (Sigma-Aldrich) was used as the standard, and the TPC was expressed as mg of gallic acid equivalents per g of extract (mg GAE/g).

#### 2.2.2. Total Flavonoid Content (TFC) Assay

Total flavonoid content (TFC) was determined based on the colorimetric method described by Nivea Moreno et al. [23] with slight modifications. A 0.1 mL aliquot of each sample was mixed with 0.9 mL of 80% ethanol. Then, 0.5 mL of this mixture was added to a test tube containing 0.1 mL of 10% aluminum nitrate (Sigma-Aldrich), 0.1 mL of 1 M potassium acetate (Sigma-Aldrich), and 4.3 mL of 80% ethanol. The reaction mixture was incubated for 40 min at RT, and the absorbance was measured at 415 nm. Quercetin (Sigma-Aldrich) was used as the reference standard, and the results were expressed as quercetin equivalents (mg QE/g extract).

### 2.3. Antioxidant Activity Assays

#### 2.3.1. 2,2-diphenyl-1-picrylhydrazyl (DPPH) Radical Scavenging Activity

The DPPH radical scavenging activity of the samples was determined according to a modified method described by Blois et al. [24]. Briefly, 160 µL of each sample at various concentrations was mixed with 40 µL of 0.15 mM DPPH solution in methanol in a 96-well microplate. The mixture was incubated in the dark at RT for 30 min. The absorbance was measured at 517 nm using a microplate reader (Bio-Rad). Methanol was used as a blank, and the DPPH solution without a sample was used as a control. The radical scavenging activity of each sample was compared based on the RC_50_ value, which is defined as the concentration required to reduce the absorbance of the control (without sample) by 50%.

#### 2.3.2. Determination of ABTS^+^ Radical Scavenging Activity

The ABTS^+^ radical scavenging activity was determined using a modified method of Re et al. [25]. A stock solution was prepared by mixing 7 mM ABTS and 2.45 mM potassium persulfate (Sigma-Aldrich) in equal volumes, and the mixture was kept in the dark at RT for 24 h to generate ABTS^+^ radicals. The resulting solution was diluted with distilled water to obtain an absorbance of 0.70 ± 0.02 at 732 nm. For the assay, 180 µL of the ABTS^+^ working solution was mixed with 20 µL of the sample solution. The mixture was incubated at RT for 1 min, and the absorbance was measured at 732 nm using a microplate reader (Bio-Rad).

#### 2.3.3. Determination of Ferric Reducing Antioxidant Power (FRAP)

The FRAP was measured using a modified method of Benzie and Strain [26]. The FRAP reagent was freshly prepared by mixing 300 mM sodium acetate buffer (pH 3.6), 10 mM 2,4,6-tripyridyl-s-triazine (TPTZ, Sigma-Aldrich) dissolved in 40 mM HCl, and 20 mM FeCl_3_ solution in a ratio of 10:1:1 (*v*/*v*/*v*) just before use. For the assay, 10 µL of the sample at various concentrations was mixed with 200 µL of the FRAP reagent in a 96-well plate. The mixture was incubated at 37 °C for 5 min, and the absorbance was measured at 593 nm using a microplate spectrophotometer (Bio-Rad). The FRAP value of each sample was calculated based on a standard calibration curve constructed with FeSO_4_·H_2_O (0–5 mM), and the results were expressed as mM FeSO_4_ equivalents per mg of sample (mM FeSO_4_ eq/mg).

### 2.4. Anti-Inflammatory Activity Assay

#### 2.4.1. Cell Culture

RAW 264.7 murine macrophages were obtained from the Korean Cell Line Bank (KCLB, Seoul, Republic of Korea). Cells were cultured in Dulbecco’s Modified Eagle Medium (DMEM; Gibco-BRL, Rockville, MD, USA) supplemented with 10% fetal bovine serum (FBS, Gibco-BRL), 100 µg/mL penicillin, and 100 µg/mL streptomycin (Gibco-BRL). Cultures were maintained at 37 °C in a humidified atmosphere with 5% CO_2_ and sub-cultured every 2–3 days.

#### 2.4.2. Cell Viability Assay

The cytotoxicity of the samples was assessed using the 3-(4,5-dimethylthiazol-2-yl)-2,5-diphenyl tetrazolium bromide (MTT) reduction assay. RAW 264.7 cells were seeded in 96-well plates at a density of 1 × 10^5^ cells/well and allowed to adhere for 24 h. After discarding the medium, cells were treated with different concentrations of the samples (0, 25, 50, 100, and 200 µg/mL) prepared in DMEM containing 100 ng/mL lipopolysaccharide (LPS; Sigma-Aldrich), and incubated for an additional 24 h under standard culture conditions. Subsequently, MTT solution (5 mg/mL) was added to each well and incubated for 4 h. After removing the supernatants, 100 µL of DMSO was added to dissolve the formazan crystals. Absorbance was measured at 550 nm using a microplate reader (Bio-Rad). Cell viability was expressed as a percentage relative to the untreated control.

#### 2.4.3. Nitric Oxide (NO) Production Assay

NO production induced by LPS stimulation was measured using the Griess reagent, based on a modified protocol described by Green et al. [27]. RAW 264.7 cells were seeded in 96-well plates at 1 × 10^5^ cells/well and incubated for 24 h. After medium removal, cells were treated with test samples at final concentrations of 0, 25, 50, 100, and 200 µg/mL in DMEM supplemented with 100 ng/mL LPS. The cells were then incubated for another 24 h at 37 °C with 5% CO_2_. Following incubation, 100 µL of the culture supernatant was mixed with an equal volume of Griess reagent (Sigma-Aldrich) in a separate 96-well plate. The reaction was allowed to proceed for 10 min at RT, and absorbance was read at 540 nm. A standard curve was generated using sodium nitrate (Sigma-Aldrich) to quantify NO levels.

#### 2.4.4. Evaluation of iNOS Expression by Western Blotting

RAW 264.7 cells were seeded in 6-well plates at a density of 1 × 10^6^ cells/well and allowed to adhere overnight. To assess the effect of *S. thunbergii* extract (collected from the coastal area of Haeundae-gu, Busan, Korea), cells were pretreated with varying concentrations of the extract (12.5, 25, 50, and 100 μg/mL) for 1 h. Inflammation was then induced by lipopolysaccharide (LPS, 100 ng/mL; Sigma-Aldrich), and incubation continued for an additional 24 h. Following treatment, cells were lysed using radioimmunoprecipitation assay (RIPA, Thermo Scientific, Rockford, IL, USA) buffer containing protease inhibitors (Thermo Scientific), and total protein concentrations were quantified using a bicinchoninic acid (BCA) assay kit. Equal amounts of protein (20 μg) were resolved on 10% SDS-polyacrylamide gels and transferred onto PVDF membranes (Millipore, Billerica, MA, USA). After blocking with 5% skim milk in TBS-T (Tris-buffered saline with 0.1% Tween-20) for 1 h, membranes were incubated overnight at 4 °C with primary antibodies specific to inducible nitric oxide synthase (iNOS; 1:1000 dilution, Cell Signaling Technology, Danvers, MA, USA) and β-actin (1:2000 dilution, Cell Signaling Technology). Secondary HRP-conjugated antibodies were applied for 1 h at RT, and signals were detected using an enhanced chemiluminescence (ECL) system (Thermo Scientific). Band intensity was measured using ImageJ software 1.54i (NIH, Bethesda, MD, USA; https://imagej.nih.gov/ij/, accessed on 15 August 2025), and iNOS expression levels were normalized to β-actin. All experiments were conducted in triplicate.

### 2.5. HPLC Analysis of Fucosterol in *S. thunbergii* Extract

High-Performance Liquid Chromatography (HPLC) analysis of fucosterol was performed using a C18 column (4.6 × 250 mm, 5 μm; ZORBAX SB-C18, Agilent, Santa Clara, CA, USA) with a flow rate of 1.0 mL/min. The mobile phase consisted of solvent A (0.1% phosphoric acid in distilled water; Sigma-Aldrich) and solvent B (acetonitrile; Sigma-Aldrich). The gradient program was as follows: 95% A and 5% B for 3 min, a linear gradient to 0% A and 100% B over 33 min, maintained for 4 min, and then returned to the initial condition (95% A, 5% B) at 40 min, followed by equilibration until 45 min. The injection volume was 30 μL of the sample solution (1 mg/mL in methanol). Detection was carried out at 230 nm using a photodiode array (PDA) detector. Linearity was confirmed across six concentration levels of standard fucosterol (0.8–193.5 μg/mL), with regression coefficients (R^2^) ranging from 0.9994 to 1.0000. The limit of detection (LOD) and limit of quantification (LOQ) were calculated as 0.12 μg/mL and 0.36 μg/mL, respectively. Accuracy was verified by recovery tests at three spiking levels (low, medium, high), which yielded recoveries of 95.1–100.4% with %RSD between 1.73 and 3.81%. Precision was assessed by repeatability and intermediate precision at 25, 50, and 75 mg levels, with an overall %RSD of 1.66%. Specificity was demonstrated by identical retention times and PDA spectral patterns between the standard and sample peaks, with no interfering peaks observed.

### 2.6. In Silico Docking of Fucosterol to iNOS

To explore the potential interaction between *S. thunbergii*-derived fucosterol and iNOS, molecular docking studies were performed using the Glide tool in Schrödinger Suite 2023-2 (Schrödinger, LLC, New York, NY, USA). The crystal structure of iNOS (PDB ID: 3E7G) was obtained from the RCSB Protein Data Bank and preprocessed using the Protein Preparation Wizard. This included optimization of hydrogen bonding networks, assignment of bond orders, and removal of non-essential water molecules. A receptor grid was generated around the native ligand-binding site. The 3D chemical structures of fucosterol were prepared using LigPrep 2.5, applying the OPLS4 force field to generate low-energy conformations. As a comparator, AT2 (ethyl 4-[(4-methylpyridin-2-yl) amino] piperidine-1-carboxylate) was included in the analysis. Each ligand was docked into the iNOS active site using standard precision (SP) mode. Docking scores (GlideScores; Schrödinger) were recorded to assess binding affinity, and molecular interactions including hydrogen bonds, π-π stacking, and salt bridges were visualized and analyzed using Maestro software v12.8 (Schrödinger 2025-4; Schrödinger).

### 2.7. Statistical Analysis

All experimental results are expressed as the mean ± standard deviation (SD) of at least three independent measurements. Statistical analysis was performed using SPSS Statistics software (version 25.0, IBM Corp., Armonk, NY, USA). One-way analysis of variance (ANOVA) was used to evaluate significant differences among groups. Post hoc comparisons were conducted using Duncan’s multiple range test at a significance level of *p* < 0.05.

## 3. Results

### 3.1. Characterization of Collection Sites and Geographical Variation

Figure 1 shows the geographical distribution of *S. thunbergii* collection sites along the coastal regions of Korea, encompassing seven ecologically distinct areas. The sampling points—Boryeong-si (West Coast), Jindo-gun (South Coast), Seogwipo-si (Jeju Island), Haeundae-gu (Southeast Coast), Sacheon-si (South Coast), Uljin-gun (East Coast), and Yangyang-gun (Northeast Coast)—represent a broad range of environmental gradients in terms of temperature, salinity, solar irradiance, and hydrodynamic conditions. All algal specimens were provided by the National Marine Biodiversity Institute of Korea (MABIK) under its biological-resource distribution program. According to Kim et al. [28]. *S. thunbergii* populations along the Korean coastline exhibit clear geographic differentiation in morphology—including thallus length, branching pattern, and holdfast size—driven by regional environmental parameters such as tidal range, wave exposure, and seawater temperature. The West Coast is typified by shallow tidal flats, high turbidity, and strong tidal amplitude, conditions that generate fluctuating salinity and light penetration. These factors can impose physiological stress but may also stimulate the accumulation of protective secondary metabolites. The South Coast, influenced by the Tsushima Warm Current, experiences higher water temperatures and nutrient enrichment, supporting rapid algal growth and metabolic activity. The East Coast is dominated by cold currents from the North Pacific, with strong wave action and pronounced seasonal variation in temperature, which are associated with slower growth but denser tissue structure. Meanwhile, Jeju Island represents a subtropical habitat with higher solar radiation and elevated seawater temperature, potentially enhancing photosynthetic efficiency and phlorotannin biosynthesis. These region-specific environmental regimes collectively shape morpho-physiological adaptation in *S. thunbergii*, which likely underlies the observed regional variation in biochemical composition and biological activity. Understanding these spatial differences provides essential ecological context for interpreting the antioxidant and anti-inflammatory outcomes presented in subsequent sections.

### 3.2. Total Polyphenol and Flavonoid Contents of S. thunbergii Extracts from Different Regions

**Figure 2:**
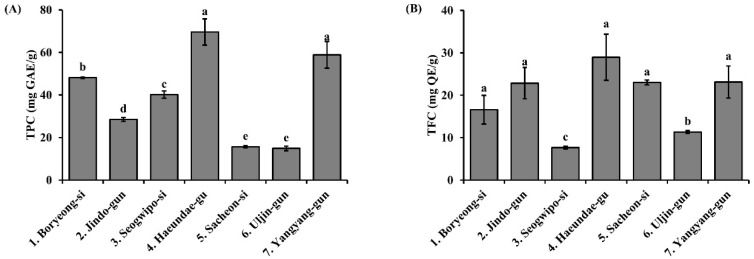
Total polyphenol content (TPC) and total flavonoid content (TFC) of *S. thunbergii* extracts from seven different coastal regions of Korea. (**A**) TPC was measured using the Folin–Ciocalteu method and expressed as mg gallic acid equivalent (GAE) per g extract. (**B**) TFC was determined using the aluminum chloride colorimetric method and expressed as mg quercetin equivalent (QE) per g extract. Data are presented as mean ± SD (*n* = 3), and different letters indicate significant differences among the regions (*p* < 0.05).

Figure 2 presents the total polyphenol content (TPC) and total flavonoid content (TFC) of *S. thunbergii* extracts from different coastal areas. The TPC values ranged from approximately 15 to 70 mg GAE/g, with the highest content observed in extracts from Haeundae-gu and Yangyang-gun. In contrast, Uljin-gun and Sacheon-si exhibited significantly lower polyphenol levels (*p* < 0.05). Similarly, TFC values varied between regions, with Haeundae-gu, Yangyang-gun, and Sacheon-si showing relatively high flavonoid contents, while Seogwipo-si and Uljin-gun exhibited the lowest levels (*p* < 0.05). These findings suggest a geographical influence on the accumulation of polyphenols and flavonoids in *S. thunbergii*.

### 3.3. Free Radical Scavenging Activity of S. thunbergii Extracts from Different Regions

**Figure 3:**
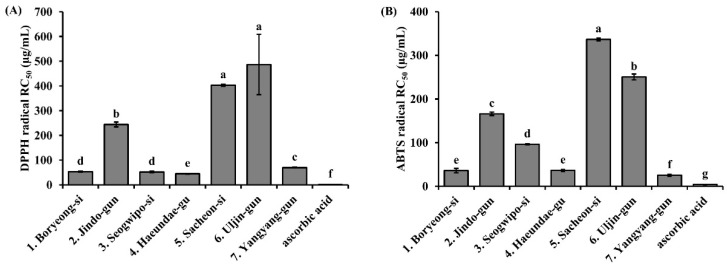
Free radical scavenging activity (RC_50_values) of *S. thunbergii* extracts collected from seven different coastal regions of Korea. (**A**) DPPH radical scavenging activity. (**B**) ABTS^+^ radical scavenging activity. Results are expressed as mean ± SD (*n* = 3). Different letters above the bars indicate statistically significant differences among samples, as determined by one-way ANOVA followed by Duncan’s multiple range test (*p* < 0.05).

The antioxidant activities of *S. thunbergii* extracts obtained from seven coastal regions were evaluated using DPPH and ABTS^+^ radical scavenging assays, and their RC_50_ (radical scavenging concentration required to reduce radical levels by 50%) values are presented in Figure 3. In the DPPH assay (Figure 3A), the Uljin-gun extract exhibited the highest RC_50_ value (*p* < 0.05), indicating the weakest radical scavenging activity among the tested samples. In contrast, the extract from Haeundae-gu showed the lowest RC_50_ value (*p* < 0.05), suggesting the strongest antioxidant capacity. In the ABTS^+^ assay (Figure 3B), a similar trend was observed. The Sacheon-si extract again showed the highest RC_50_ value, while the Boryeong-si, Haeundae-gu, and Yangyang-gun extracts demonstrated significantly lower RC_50_ values (*p* < 0.05), suggesting potent ABTS^+^ radical scavenging activities. Ascorbic acid, used as a positive control, exhibited RC_50_ values of 3.13 ± 0.02 μg/mL in the DPPH assay and 7.12 ± 0.15 μg/mL in the ABTS^+^ assay, confirming its superior radical scavenging capacity compared to all seaweed extracts. Overall, the radical scavenging activities of *S. thunbergii* extracts varied considerably depending on the geographic origin, implying that environmental factors such as water temperature, salinity, and nutrient availability may influence the bioactive compound content in these seaweeds.

### 3.4. Ferric Reducing Antioxidant Power (FRAP) of S. thunbergii Extracts from Different Regions

**Figure 4:**
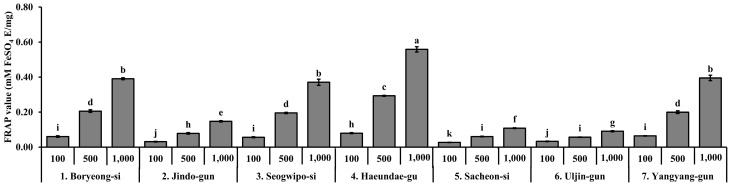
Ferric reducing antioxidant power (FRAP) of *S. thunbergii* extracts collected from seven different coastal regions in Korea at three different concentrations (100, 500, and 1000 µg/mL). Results are expressed as mM FeSO_4_ equivalents per mg of extract (mM FeSO_4_ eq/mg). Values are presented as mean ± SD (*n* = 3). Different letters indicate statistically significant differences between groups based on one-way ANOVA followed by Duncan’s multiple range test (*p* < 0.05).

The ferric reducing antioxidant power (FRAP) of *S. thunbergii* extracts was analyzed at three concentrations (100, 500, and 1000 µg/mL), and the results are shown in Figure 4. Among the tested samples, the extract from Haeundae-gu exhibited the highest FRAP value at 1000 µg/mL, significantly higher than all other regional extracts (*p* < 0.05), suggesting a superior electron-donating capacity. Boryeong-si, Seogwipo-si, and Yangyang-gun extracts also showed moderate FRAP activity in a concentration-dependent manner, particularly at higher concentrations (500 and 1000 µg/mL), with statistically distinguishable increases compared to lower concentrations. In contrast, the extracts from Jindo-gun, Sacheon-si, and Uljin-gun demonstrated relatively weak FRAP activity, with values remaining low even at the highest tested concentration. Overall, the reducing power of *S. thunbergii* extracts varied significantly by geographic origin and concentration, indicating that environmental factors may influence the antioxidant capacity through modulation of electron-donating phytochemicals such as polyphenols.

### 3.5. Cytotoxicity of S. thunbergii Extracts from Different Regions

**Figure 5:**
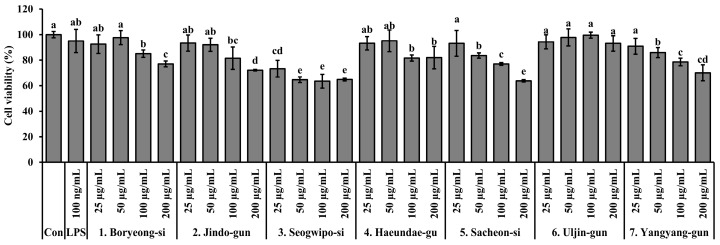
Cytotoxicity of *S. thunbergii* extracts from seven different coastal regions of Korea in RAW 264.7 macrophages treated with LPS (100 ng/mL). Cells were treated with 25, 50, 100, and 200 µg/mL of each extract for 24 h, and viability was measured using the MTT assay. Results are presented as mean ± SD (*n* = 3). Statistical analysis was performed using one-way ANOVA followed by Duncan’s multiple range test (*p* < 0.05). Different letters above the bars indicate statistically significant differences between treatment groups. Con indicates the untreated control group, and LPS indicates the lipopolysaccharide-treated group.

The cytotoxicity of *S. thunbergii* extracts from seven coastal regions was assessed in RAW 264.7 macrophages using the MTT assay following 24 h of treatment. As shown in Figure 5, most extracts exhibited low cytotoxicity, maintaining over 80% cell viability at concentrations up to 100 µg/mL. However, a notable exception was observed in the extract from Seogwipo-si, which significantly reduced cell viability even at lower concentrations (25 and 50 µg/mL) compared to the lipopolysaccharide (LPS)-treated control (*p* < 0.05), indicating relatively higher cytotoxic potential. At 200 µg/mL, additional extracts from Jindo-gun and Sacheon-si also showed a significant decrease in viability, but not as pronounced as that of the Seogwipo-si extract. In contrast, extracts from Boryeong-si, Haeundae-gu, and Uljin-gun maintained cell viability above 85% across all tested concentrations, suggesting minimal cytotoxic effects. These findings suggest that while most *S. thunbergii* extracts are safe for in vitro use up to 100 µg/mL, the Seogwipo-si sample may require caution due to its stronger cytotoxic effect at even low concentrations.

### 3.6. Inhibitory Effects of S. thunbergii Extracts from Different Regions on NO Production

**Figure 6:**
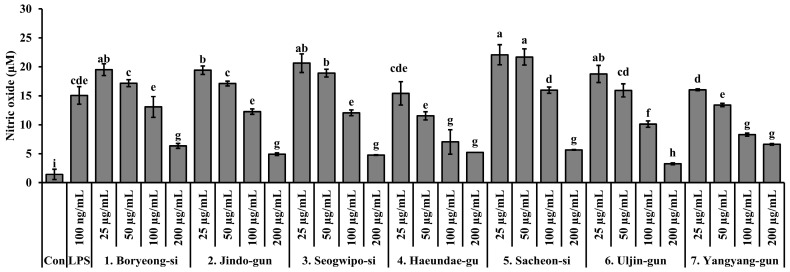
Effects of *S. thunbergii* extracts from seven different coastal regions of Korea on nitric oxide (NO) production in LPS-stimulated RAW 264.7 macrophages. Cells were treated with extract concentrations of 25, 50, and 100 µg/mL for 24 h, and nitrite levels in the culture supernatants were determined using the Griess reagent. Data are expressed as mean ± SD (*n* = 3). Different letters above the bars indicate statistically significant differences among treatment groups based on one-way ANOVA followed by Duncan’s multiple range test (*p* < 0.05). Con indicates the untreated control group, and LPS indicates the lipopolysaccharide-treated group.

The ability of *S. thunbergii* extracts from different coastal regions to inhibit nitric oxide (NO) production in LPS-stimulated RAW 264.7 macrophages is illustrated in Figure 6. All extracts exhibited concentration-dependent NO suppression, but the extent of inhibition varied significantly depending on the region of origin. Among the tested samples, the extract from Haeundae-gu demonstrated strong NO inhibitory activity even at non-cytotoxic concentrations (≤100 µg/mL), showing a statistically significant reduction in NO levels compared to the LPS-only group (*p* < 0.05). This suggests that the Haeundae-gu extract may contain potent anti-inflammatory compounds without adversely affecting cell viability. In addition, the extracts from Sacheon-si, Boryeong-si, and Yangyang-gun also showed notable NO inhibition at higher concentrations. However, some extracts, including those from Seogwipo-si and Jindo-gun, exhibited less pronounced effects and were associated with moderate cytotoxicity, particularly at higher concentrations. Taken together, the extract from Haeundae-gu stands out as a promising candidate for further anti-inflammatory evaluation due to its effective NO suppression at biologically safe concentrations.

### 3.7. Effects of S. thunbergii Extract Collected from Haeundae-gu on iNOS Expression in LPS-Stimulated RAW 264.7 Cells

**Figure 7:**
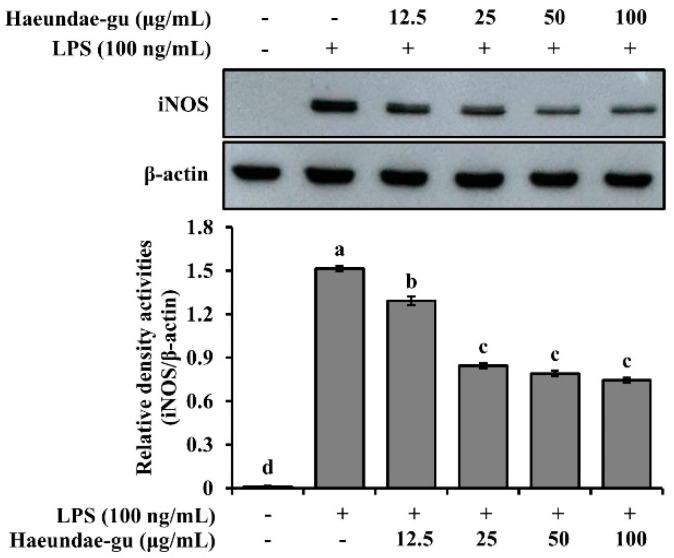
Western blot analysis of iNOS and β-actin expression in LPS-stimulated RAW 264.7 cells treated with *S. thunbergii* extract collected from Haeundae-gu (12.5–100 μg/mL). The iNOS protein levels were normalized to β-actin, and the relative intensities are shown in the bar graph. Treatment with the extract led to a concentration-dependent suppression of iNOS expression. Data are expressed as mean ± SD (*n* = 3). Different letters above the bars indicate statistically significant differences among treatment groups based on one-way ANOVA followed by Duncan’s multiple range test (*p* < 0.05).

To evaluate the anti-inflammatory potential of *S. thunbergii* collected from Haeundae-gu, we examined its effect on iNOS expression in LPS-stimulated RAW 264.7 macrophages. Western blot analysis revealed that treatment with the extract reduced iNOS protein levels in a concentration-dependent manner. As shown in Figure 7, iNOS expression was significantly decreased at concentrations of 25, 50, and 100 μg/mL, compared to the LPS-only group (*p* < 0.05). These results suggest that the *S. thunbergii* extract from Haeundae-gu effectively suppresses LPS-induced inflammatory responses by downregulating iNOS expression.

### 3.8. Identification of Fucosterol in S. thunbergii Extract from Haeundae-gu

**Figure 8:**
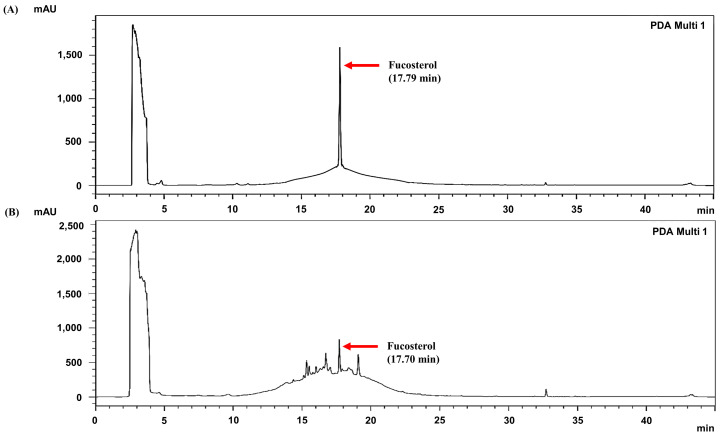
HPLC chromatograms (230 nm) of (**A**) fucosterol standard and (**B**) *S. thunbergii* extract from Haeundae-gu. Red arrows indicate the retention time of fucosterol.

The analysis of the content of the active compound, fucosterol, in the *S. thunbergii* extracts is shown in Figure 8. On the HPLC chromatogram, fucosterol exhibited a retention time of approximately 17.70 min with a content of 10.23 ± 0.17 mg/g. This quantification, together with the co-elution of the authentic standard at 17.79 min, strongly confirms the presence of fucosterol in the extracts. Fucosterol, a characteristic sterol of brown algae, is reported to possess various bioactivities, including antioxidant, anti-inflammatory, and cholesterol-lowering activities [29]. Given this fact, its presence in the Haeundae-gu extract may be a major contributing factor to the observed bioactivities of *S. thunbergii* [30].

### 3.9. Molecular Docking Analysis of Representative Fucosterol from S. thunbergii Against iNOS

To elucidate the molecular mechanism underlying the anti-inflammatory effects of *S. thunbergii*, in silico molecular docking studies were performed targeting inducible nitric oxide synthase (iNOS; PDB ID: 3E7G), a key enzyme involved in the production of nitric oxide during inflammation. Fucosterol, identified in the Haeundae-gu extract, was selected for docking simulations using the Schrödinger Glide (Schrödinger 2025-4; Schrödinger) module.

As summarized in Table 2, fucosterol exhibited a GlideScore of −4.774 kcal/mol and interacted with TYR489 through hydrogen bonding within the active site of iNOS. For comparison, the reference compound AT2 showed a stronger binding affinity with a GlideScore of −6.848 kcal/mol, forming π-π stacking with TRP194 and a salt bridge with CYS200.

**Figure 9:**
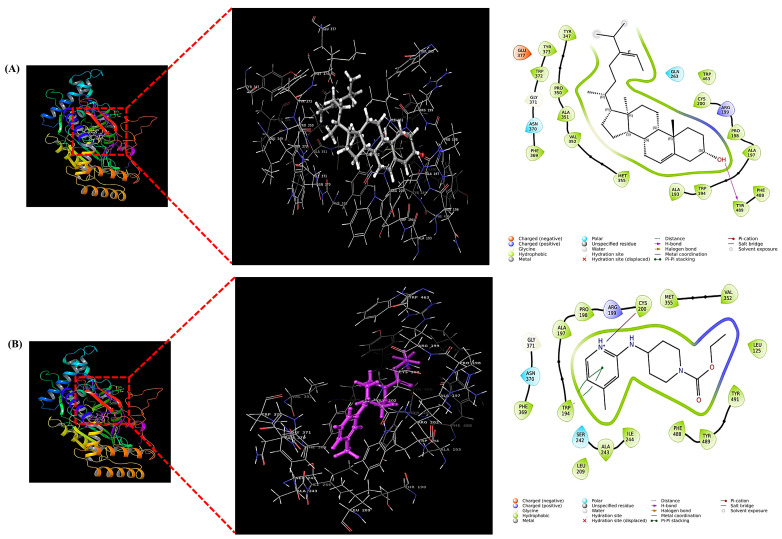
Molecular docking visualization of fucosterol (**A**) and AT2 (Ethyl 4-[(4-methylpyridin-2-yl)amino]piperidine-1-carboxylate) (**B**) within the active site of iNOS (PDB ID: 3E7G). Each panel presents the overall 3D binding pose in ribbon and surface views (**left**), an enlarged 3D view of the ligand within the binding pocket (**middle**), and the 2D ligand–residue interaction diagram (**right**). All docking simulations were performed using Schrödinger Glide Schrödinger Glide (2025-4; Schrödinger).

These results suggest that although fucosterol demonstrated a relatively moderate binding affinity compared to the synthetic inhibitor, it was still able to interact with catalytically important residues of iNOS. This finding supports the potential contribution of fucosterol to the observed in vitro anti-inflammatory activity of *S. thunbergii* extracts. Molecular interaction diagrams further illustrate the binding orientation and key residues involved in the interaction (Figure 9).

## 4. Discussion

This study provides a comprehensive multi-regional comparison of *S. thunbergii* extracts collected from seven distinct Korean coastal regions and demonstrates substantial region-specific variations in antioxidant and anti-inflammatory activities. Among these, the Haeundae-gu extract exhibited the highest total polyphenol and flavonoid contents, as well as the strongest free-radical scavenging and nitric oxide (NO) inhibitory effects. These findings highlight the significant influence of environmental and ecological conditions—including temperature, salinity, and light exposure—on the biosynthesis of phenolic and sterol-based secondary metabolites in brown algae, ultimately shaping their pharmacological potential [31,32].

Phenolic compounds, including phlorotannins and flavonoids, represent the principal antioxidant constituents in brown seaweeds [33,34,35]. The present findings revealed marked regional variations in total polyphenol (TPC) and flavonoid contents (TFC), with the highest concentrations observed in the Haeundae-gu and Yangyang-gun extracts. These compositional differences directly correlated with enhanced radical scavenging and reducing capacities, underscoring the critical role of polyphenolic abundance in determining antioxidant efficacy [36].

Quantitatively, the Haeundae-gu extract displayed superior antioxidant performance (DPPH RC_50_ = 44.54 µg/mL, ABTS RC_50_ = 36.42 µg/mL, FRAP = 0.56 mM FeSO_4_ eq/mg), exhibiting up to ninefold higher potency than the Seogwipo-si extract. These findings are consistent with previous reports on *S. fusiforme* and *Ecklonia cava*, in which polyphenol-rich fractions demonstrated comparable antioxidant capacities (TPC 40–50 mg GAE/g; FRAP 0.8–1.1 mM FeSO_4_ eq/mg) [37,38,39,40]. Similarly, other brown algae such as *Fucus vesiculosus*, *F. spiralis*, and *Ascophyllum nodosum* exhibit strong seasonal and geographical variability in phenolic content and antioxidant activity, confirming that both environmental and ecological factors play critical roles in regulating phenolic biosynthesis and antioxidant potential in brown macroalgae [41,42,43,44].

Mechanistically, the antioxidant properties of *S. thunbergii* are attributed to both hydrogen atom transfer (HAT) and single-electron transfer (SET) mechanisms, which enable efficient neutralization of reactive oxygen species (ROS) and prevent oxidative stress–mediated cellular injury [45,46,47]. These data collectively emphasize the potential of *S. thunbergii*, particularly the Haeundae-gu extract, as a potent natural antioxidant source for mitigating oxidative stress associated with chronic diseases.

Most *S. thunbergii* extracts exhibited minimal cytotoxicity in RAW 264.7 macrophages up to 100 µg/mL, except for the Seogwipo-si extract, which significantly reduced cell viability at lower concentrations (Figure 5). This localized cytotoxicity may be associated with specific environmental factors in Jeju coastal waters, such as elevated solar radiation, higher seawater temperature, or possible heavy-metal accumulation, all of which can alter metabolite composition or promote the formation of reactive compounds [48,49]. In contrast, the Haeundae-gu extract maintained excellent cell viability across all concentrations, supporting its safety profile for further anti-inflammatory evaluation.

Notably, previous studies have demonstrated that the upstream cytokine TNF-α can activate the MAPK/NF-κB/iNOS cascade, leading to enhanced iNOS expression and NO production [50]. Although TNF-α secretion was not directly measured in this study, the observed iNOS downregulation suggests that the Haeundae-gu extract may also attenuate TNF-α-mediated inflammatory signaling. This hypothesis is supported by earlier reports indicating that *Sargassum*-derived fucosterol and phlorotannins can suppress TNF-α and IL-6 expression in activated macrophages [51,52]. Future studies will therefore aim to verify whether the extract reduces secreted TNF-α levels to further clarify its upstream regulatory effects.

Functionally, the Haeundae-gu extract demonstrated strong inhibition of LPS-induced NO production without cytotoxicity, indicating effective suppression of pro-inflammatory signaling. Excessive NO generation through inducible nitric oxide synthase (iNOS) is a well-established contributor to chronic inflammation and tissue damage [53]. Western blot analysis confirmed that the Haeundae-gu extract downregulated iNOS protein expression in a concentration-dependent manner (25–100 µg/mL), indicating attenuation of inflammatory mediator synthesis (Figure 7). These results are in line with prior studies demonstrating that Sargassum-derived phlorotannins and meroterpenoids inhibit NF-κB and MAPK pathways, thereby reducing iNOS and COX-2 expression [54,55,56].

High-performance liquid chromatography (HPLC) analysis identified fucosterol as a major sterol constituent (10.23 ± 0.17 mg/g) in the Haeundae-gu extract (Figure 8). Crucially, the high content of fucosterol in the Haeundae-gu sample, compared to other regions, suggests that the specific coastal environment of Haeundae-gu may promote the biosynthesis or accumulation of lipophilic sterols, potentially due to variations in nutrient availability, water stress, or thermal exposure, similar to its effect on phenolic production. This enrichment of fucosterol is hypothesized to contribute significantly to the extract’s superior anti-inflammatory activity. Fucosterol, a predominant phytosterol in brown algae, is known for its potent antioxidant, anti-inflammatory, and cholesterol-lowering properties [51,57,58]. To explore the direct mechanism underlying this anti-inflammatory effect, molecular docking simulations revealed that fucosterol binds within the active site of iNOS through hydrogen bonding with TYR489, exhibiting a GlideScore of −4.774 kcal/mol (Figure 9). Although this affinity is weaker than that of the reference inhibitor AT2 (−6.848 kcal/mol), the interaction supports fucosterol’s mechanistic involvement in inflammation modulation through direct iNOS binding, providing a critical structural link that complements the observed cellular iNOS downregulation.

Previous studies have shown that fucosterol inhibits the production of pro-inflammatory cytokines (TNF-α, IL-6), downregulates COX-2 and iNOS expression, and activates the Nrf2 antioxidant defense pathway, thereby providing multi-modal anti-inflammatory protection [51,52]. Therefore, its elevated presence (influenced by regional variation) in the Haeundae-gu extract likely enhances and complements the biological activities of phlorotannins and flavonoids, resulting in a synergistic antioxidant and anti-inflammatory response. The integration of in vitro and in silico findings thus highlights fucosterol as a key mechanistically relevant bioactive compound contributing to the superior pharmacological efficacy of the Haeundae-gu *S. thunbergii*.

Despite the significant mechanistic insights provided by molecular docking regarding the direct binding of fucosterol to iNOS, it is essential to acknowledge the inherent limitations of this in silico approach. Docking simulations represent static snapshots and cannot fully account for the dynamic biological environment, including protein flexibility, solvent effects, and binding kinetics. Therefore, for future research, the predicted inhibitory mechanism must be strictly complemented by structural validation using methods like surface plasmon resonance (SPR) or X-ray crystallography, along with in vivo efficacy and safety assessments. Furthermore, comprehensive pharmacological profiling, including ADMET (Absorption, Distribution, Metabolism, Excretion, and Toxicity) analysis and pharmacophore modeling, will be crucial to fully establish fucosterol’s potential as a viable anti-inflammatory drug lead.

Taken together, the in vitro and in silico findings collectively indicate that the superior anti-inflammatory efficacy of the Haeundae-gu extract arises not only from its high polyphenol and flavonoid content (linked to environmental stress) but also from the mechanistically confirmed anti-inflammatory role of bioactive sterols such as fucosterol (which may also be subject to regional environmental enrichment). These integrated mechanistic and functional data provide compelling evidence supporting the potential of *S. thunbergii* from Haeundae-gu as a valuable natural resource for the development of antioxidant and anti-inflammatory nutraceuticals and therapeutic formulations.

## 5. Conclusions

This study demonstrated that *S. thunbergii* extracts exhibit distinct region-dependent antioxidant and anti-inflammatory properties, which are strongly influenced by local environmental and ecological conditions along the Korean coastline. Among the seven sampling sites, the Haeundae-gu extract contained the highest levels of total polyphenols and flavonoids and displayed the most potent free-radical scavenging and NO inhibitory activities. The superior bioactivity of this extract can be attributed to its rich phenolic composition and the presence of fucosterol, a bioactive sterol identified as a key contributor to iNOS inhibition through in silico molecular docking analysis. These findings indicate that both phenolic and sterol-derived metabolites act synergistically to mediate the antioxidant and anti-inflammatory responses of *S. thunbergii*. Collectively, this study provides mechanistic and functional evidence supporting *S. thunbergii*, particularly from the Haeundae-gu region, as a promising marine-derived resource for the development of nutraceutical and therapeutic agents targeting oxidative stress and inflammation-related disorders. Future research integrating comprehensive metabolomic profiling, environmental factor monitoring, and bioactivity correlation analysis is warranted to further elucidate the ecological determinants of metabolite biosynthesis and to facilitate the standardized and large-scale utilization of marine algal resources.

## Figures and Tables

**Table 1:** Summarizes the collection sites and extraction conditions of *S. thunbergii* collected from seven coastal regions of Korea.

No.	Collection Site	MABIK ^1^ Number	Part	Extraction Solvent	Extraction Method
1	Boryeong-si, Chungcheongnam-do	NP30210069	Whole	70% EtOH ^2^	Ultrasonic extraction
2	Jindo-gun, Jeollanam-do	NP30210018			
3	Seogwipo-si, Jeju Special Self-Governing Province	NP3022007474			
4	Haeundae-gu, Busan Metropolitan City	NP30220006			
5	Sacheon-si, Gyeongsangnam-do	NP30230130			
6	Uljin-gun, Gyeongsangbuk-do	NP30230116			
7	Yangyang-gun, Gangwon-do	NP30230178			

^1^ National Marine Biodiversity Institute of Korea. ^2^ Ethanol.

**Table 2:** Docking results of fucosterol and AT2 against iNOS (PDB ID: 3E7G).

Ligand	Glide Score (kcal/mol)	Key Binding Residues
Fucosterol	−4.774	TYR489 (H-bond)
AT2 ^1^	−6.848	TRP194 (π-π stacking), CYS200 (Salt Bridge)

^1^ Ethyl 4-[(4-methylpyridin-2-yl) amino] piperidine-1-carboxylate.

## Data Availability

Data are contained within the article.

## Abbreviations

The following abbreviations are used in this manuscript:

ABTS	2,2′-azino-bis (3-ethylbenzothiazoline-6-sulfonic acid)
AT2	Ethyl 4-[(4-methylpyridin-2-yl) amino] piperidine-1-carboxylate
BCA	Bicinchoninic acid
COX-2	Cyclooxygenase-2
DPPH	2,2-diphenyl-1-picrylhydrazyl
FRAP	Ferric reducing antioxidant power
GAE	Gallic acid equivalent
HPLC	High-performance liquid chromatography
iNOS	Inducible nitric oxide synthase
LPS	Lipopolysaccharide
MABIK	National Marine Biodiversity Institute of Korea
MAPK	Mitogen-activated protein kinase
MTT	3-(4,5-dimethylthiazol-2-yl)-2,5-diphenyl tetrazolium bromide
NF-κB	Nuclear factor kappa B
NO	Nitric oxide
ROS	Reactive oxygen species
TFC	Total flavonoid content
TPC	Total polyphenol content
